# Activity-based costing technology adoption in Australian universities

**DOI:** 10.3389/fpsyg.2023.1168955

**Published:** 2023-06-28

**Authors:** Indra Abeysekera, Rajeev Sharma

**Affiliations:** Discipline of Business and Accounting, Charles Darwin University, Darwin, NT, Australia

**Keywords:** accounting, activity-based costing, Australia, diffusion, technology, universities, social congitive theory, dynamic theory of strategy

## Abstract

Strategic cost management is vital to the Australian university sector’s performance, and activity-based costing (ABC) is a widely accepted and used accounting technology assisting with this. We examine the status of ABC technology adoption in two categories—adopters and non-adopters of the technology—informed by Technology Diffusion Framework, Social Cognitive Theory and Dynamic Theory of Strategy. The study collected data by sending a pilot-tested survey questionnaire to senior executives for electronic completion. From the 39 universities that elected to participate, 24 usable responses were received, representing a 61% response rate. The data were analyzed using cognitive characteristics supported by Social Cognitive Theory, and organizational characteristics supported by the Dynamic Theory of Strategy of universities ABC technology adopter and non-adopter status. Cognitive characteristics provide a qualitative explanation, but selected determinants show no statistical significance. Organizational characteristics also provide a qualitative explanation and show organizational revenue is the most significant determinant; followed by universities located outside the cities, and in the southern part of Australia, have more propensity for ABC technology adoption.

## 1. Introduction

Patent law is far from precise; it provides only a glimpse into the challenges of defining technology. Humans have always used tools. From early in human civilization, there has been agreement that technology is human made and not natural, primarily rooted in the concept that technology derives its meaning from tools (Agar, 2020). Technology appeared as a keyword in the 1930s, and was used in the literature in various fields including useful arts, machinery, manufacturing, industry, invention, and applied science (Schatzberg, 2006).

The patent law now ratifies that patent technology must be an innovation—not a novelty; not a discovery. The law claims that a technology must be an artifice associated with an action to be patented as a technological innovation; a human-artefact having a complex self-regulating process that maintains stability under different behavioral processes (Harwood and Eaves, 2020). The decrease in mechanical arts and the increase in applied sciences have elevated technology to the status of elite systematic knowledge (Agar, 2020).

Accounting is a versatile technology that can instruct, influence, and control people (Gomes et al., 2014; Bartocci and Natalizi, 2020). It offers various cost accounting technologies to improve organizational performance and productivity. Activity-based costing (ABC) was introduced as an innovative technology to assist firms in improving operational and financial performance by allocating overhead (indirect) costs based on activities conducted toward costing objects. Costing objects generate revenue (Vetchagool et al., 2020). Novel or more accurate approaches to overhead cost allocation have profoundly transformed accounting thought about cost management to enhance organizational performance (Quesado and Silva, 2021).

The inaccurate allocation of costs to products and services has become a growing concern for strategic decision makers (Melese et al., 2004). Inaccuracies in costing can have severe adverse consequences in the university sector, including the closing of profitable educational programs and retention of unprofitable programs. Such decisions are crucial for Australian universities, which have experienced limited growth in funding support from the Commonwealth government over an extended period, requiring universities to generate revenue. This has led universities to rely more on international students to increase student numbers by offering profitable educational programs (JRG Act, 2020; Ferguson, 2021).

The Covid-19 pandemic highlighted the importance of strategic cost management in Australian universities, which experienced a sharp fall in revenue because of astonishingly low international student enrolments, upon which universities had previously heavily depended for their revenue and organizational performance (Thatcher et al., 2020; Doidge and Doyle, 2022). Adopting innovations is in the heart of the Australian economy and was a highlight of Australian organizations successfully emerging from the Covid-19 pandemic (Abeysekera, 2023).

Given that improper costing based on activities can be very misleading in determining the cost of an educational program, it is essential that critical stakeholders properly understand ABC technology. ABC is a validated innovative accounting technology for strategic cost management (Tirol-Carmody et al., 2020). However, no systematic empirical study has investigated sector-wide ABC technology adoption in Australian universities.

This study is original because it methodologically examines the ABC technology adoption qualitatively and quantitatively. It describes and determines the influence of the cognitive characteristics of senior leaders and organizational characteristics can have on ABC technology adoption. The study is also theoretically novel. It uses three theories as its nomological net to explain ABC technology adoption using the Technology Diffusion Framework, Social Cognitive Theory, and Dynamic Theory of Strategy.

Results suggest that universities that have adopted ABC technology are mainly large in terms of revenue, and those that have not adopted it are smaller, city-based universities. Adoption continuity and organizational characteristics are crucial for sustained ABC technology adoption. The following section briefly introduces the relevant literature. Section 3 discussed the theoretical framework. Section 4 describes the data collection. Section 5 presents findings related to cognitive characteristics of senior leaders. Section 6 conducts binary logistic regression to identify variables related to organizational characteristics with a statistically significant influence on ABC technology adoption. Section 7 finishes with concluding remarks, limitations encountered, and opportunities for future research.

## 2. Relevant literature

ABC is an essential technology in accounting when firms must allocate overheads across a portfolio of products and services (Cropper and Cook, 2000). A study comparing ABC technology practices between private and public sector firms concluded there was no difference in the adoption rate (Baird, 2007). Although previous studies have provided fragmented insights into ABC technology adoption in the Australian university sector, comprehensive research is missing from the literature (Quesado and Silva, 2021).

The Australian university business model rarely has private owners, as most universities are publicly owned entities. However, universities depend on the Commonwealth government for revenue to educate domestic students and conduct research through various competitive research grant schemes. Australian universities offer students a wide range of courses and apply for competitive research funding from multiple sources. ABC technology can allocate overhead costs based on activities that generate revenue through teaching and research (Karsten et al., 2017).

These activities require accurate costing, and many overhead-causing activities make up the total cost. These include administrative costs that are indirectly rather than directly related to revenue generation through educational programs. For example, the same classroom can be used to teach several courses in an educational program. These facilities’ administrative and maintenance costs are indirectly associated with conducting classes. Academics also can teach across courses. Accurate costing of these services requires a precise approach to allocate overheads to those courses (cost objects) that generate revenue; in this regard ABC technology becomes tremendously helpful (Thatcher et al., 2020). Successfully adopting technology requires top management support (Hsu et al., 2018) having a technology champion to lead the project (Tona et al., 2016), and technical expertise (Gabryelczyk, 2019). A longer time to implement and receive intended benefits can deter organizations from technology adoption (Bunduchi et al., 2011).

Rogers and Shoemaker (1971) were early authors explaining technology diffusion, such as ABC. They described technology diffusion as the process whereby an idea or innovation is communicated through the social system over time. It is this diffusion that translates an innovative technology into productive use. However the diffusion adoption pattern is not homogenous: early authors suggested it follows a typical S-shaped curve over time because several factors contribute to firms adopting technology diffusion differently. Senior leaders’ cognitive and organizational characteristics of universities are two dimensions of technology adoption (Das, 2022).

## 3. Theoretical frameworks

### 3.1. Technology diffusion framework

**Figure 1:**
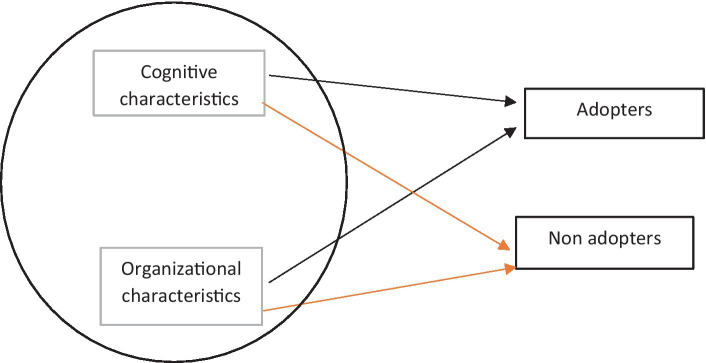
The relationships between Technology Diffusion Framework strands and ABC technology adoption outcome.

A study that reviewed the theoretical perspectives and empirical evidence around diffusion of technological innovation in the literature and found numerous differences in theoretical approaches to diffusion of technology. The review concluded that diffusion follows a complex process, and multiple factors contribute to adoption or non-adoption of technology (Das, 2022). Figure 1 shows the two theoretical strands synthesized and aimed to provide reasons for technology adoption and non-adoption.

There are two strands as drivers in a technology diffusion framework. The first strand is about the cognitive characteristics of technology adoption; the second strand is about organizational characteristics (Das, 2022). The theoretical framework can account for the technology adoption phenomenon with these two descriptive strands (Nilsen, 2015).

### 3.2. Social cognitive theory

The Social Cognitive Theory states that two aspects influence technology adoption and diffusion. The first is that those deciding about technology adoption can learn through their capabilities and experiences, as well as from others. These learnings guide senior leaders about how important such adoption choices are to them. The second aspect is a senior leader’s belief about their capabilities (self-efficacy) in adopting a specific project, such as ABC technology, from start to completion. Psychological and affective states influence these attributes (Bandura, 1986; Straub, 2009; Talukder, 2012). Based on the Social Cognitive Theory, senior executives can play a crucial role in ABC technology adoption and non-adoption through their capabilities, experiences and self-efficacy.

**Figure 2:**
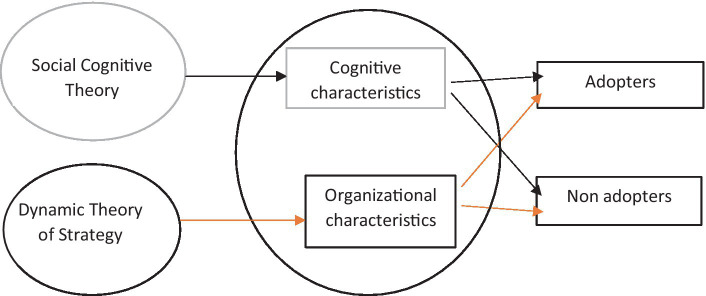
The nomological net of theoretical frameworks for ABC technology adoption.

Figure 2 shows the nomological net of the Technology Diffusion Framework and Social Cognitive Theory and the connection between Social Cognitive Theory and its connection to the two strands in the Technology Diffusion Framework. The Social Cognitive Theory helps to understand ABC technology adoption in terms of cognitive characteristics (Roberts et al., 2021).

There are cognitive differences between men and women in making technology adoption decisions. The attitude towards adopting technology drives men, and norms and perceived behavioral controls drive women (Venkatesh et al., 2000). Technology adoption can associate with the anxiety of failing to adopt successfully; senior leaders having previous experience and technical knowledge by gaining qualifications can decrease perceived anxiety and increase adoption (Roberts et al., 2021). Older adults are less likely than younger adults to adopt technology because of the differences in cognitive abilities, technology self-efficacy and technology anxiety (Czaja et al., 2006). The study examines these aspects by stating the following research question (RQ):

RQ1: what cognitive characteristics contribute to adopting or non-adopting ABC technology?

### 3.3. Dynamic theory of strategy

Resource availability in the firm is a vital organizational characteristic (Ghobakhloo et al., 2012). However, each university responds strategically differently to its organizational environment considering its contextual situation, which can include its geographical location and mix of student composition such as domestic versus international students (Skiti, 2020).

The Dynamic Theory of Strategy shows that the extent of a firm’s success or failure depends on strategy (course of action). Each firm differs from its industry peers because of how it responds to its situational position to sustain its advantage. The decisions made by senior leaders determine the drivers of undertaking organizational activities, to have a sustainable competitive advantage toward attaining its goals (Porter, 1991). These include getting internal functional areas to work together and aligning the organization’s strengths and weaknesses to achieve organizational aims such as maximizing revenue (Porter, 1991).

Strategy research focuses on investigating the reasons behind organizations becoming successful. Researchers examine various success outcomes with determinants. Success is about attaining a competitive position (Porter, 1991). In this study, ABC technology adoption is the outcome. Research can investigate strategy as a static phenomenon at a given time or a dynamic phenomenon sustained over time. The industry sectors can differ in their success because of the industry characteristics, such as the university sector. Still, universities can vary in decisions about sustained ABC technology adoption (Van den Bosch, 1997, 91–100).

Figure 2 shows the nomological net of the Technology Diffusion Framework and Dynamic Theory of Strategy. The Dynamic Theory of Strategy informs the general diffusion of theoretical strand institutional characteristics.

The organizational characteristics comprise firm size (by revenue, organizational size by staff numbers), and research shows a positive relationship between organizational size and technology adoption (Trigo et al., 2015). Research shows a firm’s brand image is positively associated with technology adoption (Corkindale and Belder, 2009) the university’s prestige as a Group of eight (Go8) Universities in Australia. The third and fourth organizational characteristics relate to the university’s geographical location (city-based or regional, southern state or any other). Research shows geographic location can influence technology adoption (OECD/Eurostat, 2019). The study examines the organizational characteristics, stating the following research question (RQ):

RQ2: what organizational characteristics contribute to adopting and non-adopting ABC technology?

## 4. Methodology

### 4.1. Data sources

An electronic questionnaire prepared in English generated the primary data; Australian universities listed by the Australian Education Network. There were 39 universities agreed to participate in the study.

### 4.2. Survey questionnaire structure

The questionnaire developed for this research included both quantitative and qualitative questions, allowing respondents to include qualitative responses (open questions used response text boxes to allow written answers). The study administered the questionnaire survey electronically, reaching geographically dispersed respondent universities at relatively low cost in the large island country of Australia (Wilkinson et al., 2020).

We chose senior leaders as respondents to the questionnaire as they are the key decision makers regarding ABC technology adoption. The questionnaire underwent pre-testing to ensure simplicity of the questionnaire’s language and adequacy of instructions. Questions followed a non-uniform response pattern to enhance the data collected. The questionnaire provided detailed instructions to all respondents to explain key research issues and minimize non-response.

There are several causes of common method bias. The data collection method can introduce measurement error when the same questionnaire includes independent and outcome variable items. The outcome variable explained in this study was not pre-determined but arose from analyzing the questionnaire data. The questionnaire had 71 questions, and the scale length reduced access to previous responses, which prevented participants from using short-term memory as a guide to answering new questions, and participants were unable to scroll back up to revisit the answered questions (Kock et al., 2021). The study took steps to mitigate measurement bias by undertaking all procedural remedies at the questionnaire design stage, where respondents answer questions relevant to them only.

No single respondent had access to all 71 questions because each respondent only had the parts relevant to their ABC adoption status. The survey questionnaire had five parts. Part 1: information about voluntary participation and Part 2: demographic questions. They were provided to all respondents. Part 3: adopters of ABC; Part 4: tried and not adopted ABC; and Part 5: non-adopters of ABC. These parts were specific to the university’s technology adoption or non-adoption, and participants accordingly chose the relevant part to complete. The survey was marked as completed when the respondent had answered the last question of Part 3, 4, or 5. The survey then directed the respondent to a final question asking if they wanted to receive a copy of the research abstract.

Each question item in Parts 3, 4, and 5 had a textbox area where respondents could type their comments. They served as qualitative responses using the thematic analysis approach to uncover descriptors using latent meanings contained in the respondents’ comments, guided by the theories used in the study. The theoretical assumptions in the theories set the boundaries for researcher reflections, minimizing bias (inaccuracies) of interpretations. There are several ways to conduct thematic analysis, and they fall into two streams; scientifically descriptive and artfully interpretive; this study used the former approach (Braun and Clarke, 2023). Developing descriptors required going through several iterations of respondents comments. The primary researcher engaged in such iterations inductively, deriving and reflecting upon the meaning (Trainor and Bundon, 2021).

### 4.3. Pilot testing of the survey questionnaire

The survey questionnaire was pilot tested for ease of use (face validity), and ease of understanding the questions (content validity). The questions solicited responses using a binary or Likert scale regarding the completeness, length of time to complete the survey, quality of responses for statistical analysis, order bias, and ethical considerations. These feedback provided researchers to ascertain the accuracy of measuring factors contributing to ABC technology adoption (criterion validity) (Boateng et al., 2018). A small group of academics not involved in the study having experience in conducting survey questionnaire attempted the questionnaire twice with an interval of 2 to 3 weeks, exhibiting a high test–retest correlation measure indicating item reliability.

The pilot testing of the survey questionnaire aimed to identify problems with the survey structure and data collection protocols (Teddlie and Tashakkori, 2009). Ten participants completed the electronically provided pilot questionnaire. Seven were accountants, of whom four were PhD CPAs, and three were CPAs with an MBA or Bachelor of Accounting degree. Three were non-accounting PhDs with experience with surveys and research. Their feedback led to elimination of several questions, improved clarity of questions, avoided duplication, and changed the order of several questions.

### 4.4. Response rates

The study sent multiple reminders to participants to maintain contact and explain the importance of their participation. The survey questionnaire instrument used in this study required a reasonable voluntary time commitment from the chief financial officer (CFO) or equivalent listed in the Australian University Network. The study contacted potential respondents before emailing the URL for the survey questionnaire to increase the response rate (Menon and Muraleedharan, 2020).

We sent email requests that included a URL to the survey questionnaire to 39 Australian universities, 27 of which responded to the questionnaire within the specified period, giving an initial response rate of 69% (*n* = 27/39). Scrutiny of the responses showed that three surveys were incomplete. They were thus deemed unsuitable for analysis, and excluded from the overall data analysis, providing an effective response rate of 61% (*n* = 24/39).

This response rate compares favorably with similar studies in Australia and overseas, particularly with Baird’s (2007) study covering several sectors including universities, which had an overall response rate of 46% and a sectoral response rate of 56% for universities.

### 4.5. Sample representativeness

As shown in Table 1, the study investigated whether the sample represents the Australian University population by investigating the parameters and conducting chi-square tests for statistical significance that compared the sample and the population. The study examined three parameters. First is whether the university is based in a city or in the region. The second is whether it is a Go8 university or other. Third is whether the university is located in a southern part or another part of Australia.

**Table 1:** Sample representativeness of the population.

Parameter	Sample	Population	*χ*^2^ (value of *p*)	Yates corrected *χ*^2^ (value of *p*)
City-based versus regional	13 versus 9	22 versus 20	0.02 (0.89)	0.014 (0.91)
G O 8 versus other	5 versus 19	8 versus 34	0.031 (0.86)	0.021 (0.88)
Southern versus other	15 versus 9	28 versus 14	0.117 (0.73)	0.005 (0.94)

The study conducted a 2 × 2 chi-square statistic comparing the sample and population. The study also used Yates correction for two dichotomous parameters to rectify deviations from the theoretical or smooth probability distributions (Giannini, 2005, 142–173). The results confirm that the sample represents the population, showing no statistically significant difference between the three parameters. The study could not compare the revenue and staff numbers of the sample with the population because the participants took part in the survey anonymously. Further, the survey asked participants to report them within specified categorical ranges rather than as absolute numbers. Identifying the exact University was not possible because of their anonymous participation in the survey.

### 4.6. Descriptive statistics

Table 2 summarizes the descriptive statistics. The sample shows that 63 per cent of universities earn annual revenue over 500 million. In terms of organizational characteristics, 29 per cent of universities have staff numbers over 2,000. 54 per cent of the universities are located in a city of Australia. 63 per cent of universiites are located in a southern part of Australia. They are New South Wales, Victoria, South Australia, Australian Capital Terrritory, and Tasmania. 21 per cent of universities in the sample are in the Go8.

**Table 2:** Descriptive statistics of the sample.

Variable	Variable values as the number of observations
*Outcome*	
ABC adoption	yes (=24); no (=9)
*Cognitive*	
Gender	Male (=13), Female (=11)
Highest qualification	Bachelor (=10), Master (=14)
Accounting experience in years	<10(=1), 10 to15(=1), >15 to 20(=6), >20(=16)
Current place experience in years	0 to 5(=13), >5 to 10(=9), >10(=2)
Participant age in years	30 to 39(=4), 40 to 49(=14), 50 to 59(=6)
*Organizational*	
Revenue	≥Annual revenue Australian $500 million, large (=16); otherwise small (=8)
Staff	>Staff numbers 2,000, large (=5); otherwise small (=19)
City-based	Located in a city (=13); located in a regional area (=11)
Southern part	Located in the southern part of Australia (=15); other (=9)
Go8	Go8 university (=5); other university (=19)

In terms of cognitive characteristics of participants, 54 per cent of them were males. 58 per cent reported a master’s degree as their highest qualification. 25 per cent had more than 20 years of accounting related work experience. 54 per cent had less than 5 years experience working at the current university. They indicate that participants had substantial work knowledge but limited organization-specific knowledge. 58 per cent of participants reported their age as between 40 and 49 years.

### 4.7. Data analysis approach

Respondents provided qualitative and quantitative data through the survey questionnaire. The study analyzed qualitative data to discover descriptors related to Social Cognitive Theory and Dynamic Theory of Strategy. The literature has provided variables related to technology adoption and non-adoption, and the study identified them under those relating to Social Cognitive Theory and Dynamic Theory of Strategy. The study empirically tested those variables as determinants of ABC technology adoption and non-adoption. Section 5 presents qualitative and quantitative findings relating to Social Cognitive Theory. Section 6 presents qualitative and quantitative results of the Dynamic Theory of Strategy.

## 5. Findings on cognitive characteristics

This section presents the qualitative findings and quantitative findings relating to cognitive characteristics.

### 5.1. Qualitative findings

#### 5.1.1. Adopters

Most universities in the sample used ABC technology, with 15 respondents (62.5%) identifying themselves as “adopters,” confirming present use. This percentage is significantly higher than the ABC adoption reported for Australian Universities in the past (Baird, 2007). It implies that technology adoption occurs over time in the face of increased knowledge/familiarity, persuasion, decision maker inputs, implementation, and later confirmation that it is a sound technology to use (Das, 2022). Table 3 summarizes the qualitative cognitive reasons for adoption provided by the study respondents.

**Table 3:** Qualitative reasons by cognitive characteristics for ABC technology adoption.

Outcome category	Findings summary
Adopters	Top management support Appointing a project champion to lead the technology adoption project Benefits accruing from adoption
Non adopters	Top management had fewer years of work experience at the university No one led the ABC technology project They perceived the costs do not justify the benefits of adoption

The results identified the contribution of ABC technology in supporting “external reporting requirements” as a critical reason for adopting ABC technology. This response was consistent with the strategic orientation of Australian universities operating in a complex and unpredictable macro environment (Holbrook and Sanberg, 2013). Two respondents extended this to the specific requirements of schools and departments within their university to facilitate unit-specific decisions.

In terms of the qualitative reasons for adopting ABC technology, 50% (12/24) of the respondents stated that the primary motivation was to support the need for the senior leaders to have better access to information for strategic decision making.

More prominent universities regularly received external funding from other external stakeholders (Rouse et al., 2018). According to one respondent in such a university, as part of reporting and acquittal, many external fund providers required specific costing details and information, which were better enabled by ABC technology (JRG Act, 2020; Ferguson, 2021).

#### 5.1.2. Non-adopters

Nine of 24 universrites (37%) did not adopt it. Table 3 summarizes the reasons for not adopting ABC technology. There were two groups, those never trialled, others trialled and did not adopt. They did not see ABC as a sustainable technology for continued use. Their institutions also presented cognitive and organizational barriers to adoption.

The individual respondents for non-adopter universities had formal qualifications that suited their current positions (five of seven had a master-level qualification). They also had accounting work experience, with six of seven respondents having over 20 years of accounting experience. These indicate these attributes did not qualitatively play a substantial role in ABC adoption.

These senior executives had limited organization-specific knowlege in their current position. Participants from all seven universities had less than 4 years of experience in their present job. Research suggest that limited organization-specific knowledge can make goal setting difficult, such as implementing the ABC technology, which requires detailed thinking in the organizational context (Lee et al., 2015).

The qualitative data highlighted two factors that led to non-adoption. The dominant view reflected a lack of top management support and an indifferent attitude toward ABC technology as a reason for not adopting it as a cost management technology (Hsu et al., 2018; Ahmed and Philbin, 2022); understandably, no one in the non-adopter category identified as an ABC champion (Tona et al., 2016).

### 5.2. Quantitative findings

The rule of thumb for logistical regression is to have ten observations representing one variable so each variable can have sample observations with the least frequent outcome (Pal, 2021). According to the agreed-upon rule, the model can overestimate variables as significant by moving away from the null hypothesis of a no-statistically significant relationship. However, research indicates it is an empirically weak rule (Van Smeden et al., 2016).

Table 4 shows the demographic determinants investigated in ABC technology adoption. The study used binary logistic regression using determinants collectively and individually; found that models relating to those investigations were not statistically significant.

**Table 4:** Cognitive determinants of ABC adoption using binary logistical regression.

Variable	Attribute
Gender	Male = 0, Female = 1
Qualifications	Bachelor’s degree = 1, Master’s degree = 2
Accounting work experience	less than 10 years = 1, 10 to 15 years = 2, over 15 years to 20 years = 3, and over 20 years = 4
Current place work experience	Less than 5 years = 1, 5 to 10 years = 2, and over 10 years = 3
Participant age	30 to 39 years = 1, 40 to 49 years = 2, and 50 to 59 years = 3

## 6. Findings on organizational characteristics

### 6.1. Qualitative findings

#### 6.1.1. Adopters

Table 5 summarizes the qualitative findings. There was little difference in geographic context (city vs. regional base) for the adopter sample, which consisted of nine city-based and eight regional universities. However, universities that have adopted ABC technology associated with higher annual turnover.

**Table 5:** Qualitative findings by organizational characteristics for ABC technology adoption.

Outcome category	Findings summary
Adopters	Using the technology for diverse costing applications Large universities (Go8 and IRU) by revenue and staff number bracket
Non adopters	They are small city-based universities Have lower revenue and staff numbers It takes a longer time to implement

The IRU (Innovative Research University group) and Go8 university categories dominated ABC technology adopters. Four of five IRU and three of five Go8 were adopters, confirming that the continued use of ABC technology for more prominent research intensive universities was acceptable and valuable for overhead cost allocation (Holbrook and Sanberg, 2013).

Training of staff, particularly those in the accounting and finance sections (and their equivalent business units) before full-scale adoption of ABC technology was a significant contributing factor reported by the cohort of adopters (Lukowski et al., 2021). Respondents stated that the quality of training given to end users of both ABC and accounting was a relevant factor, with five institutions communicating outstanding success, nine stating moderate success, and one reporting moderate lack of success.

Large universities by revenue base, found the ABC technology innovative enough to keep using it. 14 of the 16 large universities (annual revenue over 500 million) have adopted ABC technology. Those universities encountered funding pressures exerted by the Commonwealth government that forced them to adopt ABC technology to assign overheads to cost objects, thus determining their profitability more accurately (Parker et al., 2023).

#### 6.1.2. Non adopters

Of the nine universities that have not adopted ABC technology, five were from the southern states, and four of them were small by revenue. Three of the four small non-adopter southern states universities had city locations. There were five regional universities and only one regional university, which was a small by revenue that had not adopted ABC technology. Six universities with large staff numbers have not adopted ABC technology indicating universities with large staff numbers showed no positive association. Universities large by revenue are more financially progressive. They are likely to adopt ABC technology, and universities with large staff numbers are more socially progressive and do not consider ABC technology with similar merit.

There were two universities trialled and did not adopt it and seven universities did not consider it. Ahmed and Philbin (2022) identified “top management support” as a crucial factor in successful ABC technology adoption. The most cited reason for non-adoption was the cost and time required (Bunduchi et al., 2011), followed by a shortage of qualified staff to implement the new ABC technology (Gabryelczyk, 2019).

### 6.2. Empirical model for organizational characteristics

The literature led to identification of variables relating to the Dynamic Theory of Strategy. The logistic regression model uses the maximum likelihood technique for variable parameter value estimation while simultaneously including all multiple variables (Pal, 2021). As noted for the empirical model for cognitive characteristics, the rule of thumb for logistical regression is to have 10 observations representing one variable so that each variable can have sample observations with the least frequent outcome (Pal, 2021). Our empirical model has five variables with 24 observations. According to the agreed-upon rule above, a model can overestimate variables as significant by moving away from the null hypothesis of no statistically significant relationship. However, research shows this is an empirically weak rule (Van Smeden et al., 2016).

Table 6 summarizes the variable selection. The study conducted binomial logistic regression using ABC technology adopters (=1) and non-adopters (=0) as the outcome variable representing the strategy construct. The model includes five determinant variables. It examined the influence of university size by revenue with $500 million or more per year as large (=1); and others (=0). The dichotomous size classification is contextual and reflects the typical Australian university size. Another variable indicative of size is the number of employees: universities with more than 2,000 employees are considered large (=1); otherwise small (=0). Revenue and staff number variables represent the size construct. The third variable is whether the university was based in a city (=1) or a regional area (=0) in Australia. The fourth variable is whether the university was established in the southern part of Australia (=0) or elsewhere (=1). The latter two variables represent the geography construct. The fifth variable is prestige: Go8 universities, the leading research-intensive universities in Australia (=1); and others (=0).

**Table 6:** Variable selection for the binary regression model.

Construct	Variable	Variable measurement
*Outcome*		
Strategy	ABC adoption	ABC technology adopter (=1); non-adopter (=0)
*Organizational determinants*		
Size	Annual revenue	≥Australian $500 million, large (=1); otherwise small (=0)
Size	Staff numbers	>2,000, large (=1); otherwise small (=0)
Geography	City based	City (=1); regional area (=0)
Geography	Southern state	Southern part of Australia (=0); other (=1)
Prestige	Go8	Go8 (=1); other (=0)

Table 7 summarizes the regression results. The study ran the binary logistic model to obtain regression coefficients using the robust errors function because violating uniform variance (homoscedasticity) can affect the determinant variable parameter values. Heteroscedasticity can arise because the determinants and outcome variables have a non-linear relationship.

**Table 7:** Organizational determinants of ABC adoption using binary logistical regression.

ABC adoption	Coefficient	Robust standard error	*Z* value	Probability |*z*|	Lower confidence	Upper confidence
Revenue	18.99^***^	1.53	12.4	0.000	15.98	22.00
Staff size	−16.98^***^	0.76	−22.5	0.000	−18.46	−15.49
City based	−2.71^**^	1.39	−2.0	0.050	−5.43	0.00
Southern	2.35^**^	1.18	2.0	0.046	0.04	4.65
Go8	−0.78	1.54	−0.5	0.610	−3.79	2.23

Statistical significance: ^*^10% or less, ^**^5% or less, and ^***^1% or less.

Correcting for non-linear effects in the model is crucial; not doing so can affect the parameter values and standard errors of determinant variables in the model. Table 7 reports the findings measuring confidence at 95%. The number of observations is 24. The log pseudo-likelihood is −9.24, The probability of chi-square being greater than 2 is 0.001 showing it is a statistically significant model, and the Wald chi-square 2 (5) = 511.45. The binary logistic model results for ABC technology adoption (=1) and non-adoption (=0) are as follows.

Revenue had a substantial influence on ABC technology adoption; a university getting into over 500 million Australian dollars of annual revenue category increases the propensitity of that University adopting ABC technology by 18.99 times. Staff size was a significant negative determinant. The University employs over 2,000 staff compared to employing less than 2,000 staff, can decrease the ABC technology adoption propensity by 16.98 times. The negative coefficient for the relationship with city based variable showed that regional universities had a 2.71 times propensity for ABC technology adoption. Being a university in a southern part of Australia was associated with a 2.35 times propensity compared to a university located in other parts of Australia, to adopt ABC technology. Being a prestigious university, as a Go8 university, had no statistical significant influence on ABC adoption.

## 7. Final remarks

Table 8 summarizes the findings of the study. The qualitative analysis confirmed the importance of top management commitment and appointing a project lead champion to make ABC adoption. The adoption must benefit the organization more than the cost incurred in investment and ongoing operation.

**Table 8:** Summary of findings of ABC technology adoption.

Characteristics	Adoption descriptors	Non-adoption descriptors
Cognitive findings	Top management support crucial, Project champion can more effectively lead the project, There are perceived economic benefits of adoption	Not significant determinants: Gender, Qualifications, Accounting work experience, Current place work experience, Participants age
Organizational findings	Size, Geographic location, University prestige	Determinants significantly influencing are: 1) Annual revenue positively 2) Staff size negatively 3) City-based location negatively 4) University located in the Southern part of Australia positively

The study uncovered qualitative descriptors for the cognitive behaviors on adoption and non adoption that conform with the Social Cognitive Theory. However, senior executives cognitive characteristics obtained from the literature as relevant to technology adoption and tested through the empirical model did not stasistically significantly influence ABC technology adoption. The study also uncovered qualitative descriptors for organizational characteristics. The binary regression model that tested variables obtained from the literature as relevant to technology adoption showed that large universities by revenue, universities located outside cities, and universities in the southern part of Australia are statistically significant with more propensity in ABC technology adoption.

Australian universities are crucial in the Australian socioeconomic fabric as an export earner, a disseminator of new ideas and knowledge, and a skill builder for productivity growth (Deloitte Access Economics, 2020). As predominantly publicly funded institutions, stakeholders expect universities to operate in a social, responsible, and financially prudent manner. Our findings show that innovative accounting technology assumes new meaning in the highly competitive market for global education. New accounting technology can improve decision making accuracy and become an object of legitimization in the eyes of prominent stakeholders.

The results show that ABC technology is an accounting innovation only recently adopted by Australian universities; except for two respondents, users of ABC technology had implemented it within the previous decade. This period coincides with the timeline following the 2008 global financial crisis (GFC), when Australian universities were reviewing their strategic options, including cost reduction and process improvement. Financial stringency characterized the post-GFC era, and Australian universities were not immune to its downside effects. Many universities experienced a decline in international student enrolments at this time, adding to the financial pressure they were experiencing. Although international student numbers are increasing although the Covid-19 pandemic is easing but far from over (The Lancet, 2023), stringent funding continues to be offered to Australian universities by the Commonwealth government (Molla and Cuthbert, 2022; Parker et al., 2023).

Australian universities have adopted ABC technology because it enhances process efficiency through accurate overhead cost allocation to revenue-generating cost objects. It is a widely validated best practice where universities can convince crucial stakeholders about using good financial management practice. The Covid-19 pandemic provided another reminder about the necessity for efficient and effective cost allocation, as new standards set since the pandemic may bring different challenges. These include the volume and type of international students, their income streams, the courses that gain more popularity, and additional research streams applicable to university performance (Thatcher et al., 2020). Next, we show how the findings of this study have organizational, political, and theoretical implications.

### 7.1. Organizational implications

The study points to ABC technology adoption can gain wider acceptance with the support of the senior leadership at universities. Once adopted, ABC technology is likely to benefit academic and non-academic university processes. Top management support for ABC technology varied significantly and influenced the institutional choice in favor of ABC technology. Decisions include fair resource allocation to functional academic units within the university, restructuring them or their activities, and identifying duplicated non-value-added activities.

None of the universities reported reasons such as “costing inaccuracy” for non-adoption. The primary data point only to “strategic decision making” as a critical driver of ABC adoption in Australian universities. From the university’s perspective, the system also appears to be a source of earning legitimacy from stakeholders’ perspective for good financial management.

Training in the design and adoption of ABC technology was seen as a meaningful way to integrate ABC technology into university strategy, performance evaluation, and compensation schemes. The universities that have implemented ABC technology were committed to staff training; pre-implementation training provided a mechanism for employees to understand and accept ABC technology and to feel comfortable with it.

Consistent with contemporary economic and political reality, the study argues that it is appropriate for universities to consider adopting and implementing ABC technology to efficiently improve organizational resources and effectively navigate product complexity. Complex organizations like universities are likely to have much higher indirect and support costs (more overheads than direct costs) because of their diverse product mix and complex processes (Morgan and Daniels, 2001). Therefore, they are more likely to benefit from ABC technology.

The research findings provide valuable insights for understanding the strategic role of senior leadership in facilitating the transition from a traditional costing and cost management approach to an ABC technology. As an accounting technology enabling a strategic vision, ABC is appropriate for current non-adopters to embrace as an enabling technology.

### 7.2. Political implications

The funding criteria imposed on Australian universities by the Commonwealth government constantly change. Universities receive government funding based on full-time-equivalent domestic students taking up Commonwealth-supported places. Each such university placement attracts a government grant, but the amount received depends on the field of education. Although universities continue to receive funding based on student placements, the Commonwealth government has capped this funding at the 2017 level. Since 2020, the cap has risen by a small amount based on a performance funding model, requiring production of job-ready graduates (JRG Act, 2020; Ferguson, 2021).

### 7.3. Theoretical implications

The results support the two strands in the Technology Diffusion Framework (Das, 2022), the Social Cognitive Theory (Bandura, 1986; Straub, 2009), and the Dynamic Theory of Strategy (Porter, 1991). The nomological net facilitates us in understanding adoption and non-adoption. Oragnizational characteristics also help us appreciate that changes to the formal structures of universities over time can influence changes to ABC technology adoption decisions. The Commonwealth government is a crucial stakeholder that can facilitate ABC technology adoption, requiring universities to demonstrate sound strategic cost management to obtain public funding.

### 7.4. Limitations and future research

Bias is a systematic error that can decrease the validity of research findings. It can systematically enter into the study in the design, conduct, and analysis; it can arise through two sources. First, the selection bias, such as selecting respondents, and the second is information bias such as respondents self-reporting through a survey questionnaire. There are four aspects to information bias. First, is the social desirability bias, where respondents provide socially desirable responses to private (such as income) or socially sensitive (such as drug use) topics, despite maintaining their anonymity. Recall bias is the second aspect where respondents making errors in recalling information to report. Measurement bias is the third aspect that can arise from instrument inaccuracies. Confirmation bias is the fourth aspect, where respondents respond based on their pre-conceptions, beliefs, and preferences (Althubaiti, 2016).

Research studies often overlook these systematic biases in data acquisition that can decrease validity or purposefully accommodate them can increase the validity of findings. For instance, this study purposefully introduced confirmation bias to enhance validity of qualitatively obtained respondents’ cognitive attributes. Respondents were able to make comments that supplemented survey questionnaire item responses. The study further improved the accuracy by cross-checking whether comments were consistent with item responses to the questionnaire, such as positive questionnaire response is accompanied by positive comments, and vice versa (Althubaiti, 2016).

However, other aspects of systematic can decrease valid findings. Selecting senior leaders of the universities as respondents, pilot testing the questionnaire, and making respondents to complete only the parts relevant to them in the questionnaire were aimed to counter such effects. Although the study cannot guarantee that it eliminated all the bias, future studies adhering to standardized best practice guidelines will be helpful (Boateng et al., 2018). They can triangulate quantitative and qualitative methodologies to increase data validity (Campbell et al., 2018).

Universities can be adopters or non-adopters of ABC technology. Almost one-third of the invited respondents did not participate in this study, limiting generalization of the results to the entire Australian university sector. Although the study showed that the sample represents the population, there is still a margin of error about the proportion of adopters to non-adopters. The study could not compare the representativeness of cognitive characteristics in the sample to the population because they are not publicly available data. Further, the sample of 24 universities was inadequate for a questionnaire with 71 items to conduct diverse statistical analyses. Future research could increase the sample size by soliciting at least five participants from each university to increase representativeness, given the fixed number of Australian universities. The future study can entice all universities to take part in the study.

The literature reports numerous reasons for not adopting ABC technology. Some common causes are (1) satisfaction with an existing cost accounting system (Pavlatos and Paggios, 2009); (2) the high perceived cost of initial ABC technology adoption (Simmons et al., 2006); (3) lack of adequate training in ABC technology implementation; and (4) lack of time to assess ABC technology suitability for their university. Future research might extensively explore these, identifying as determinants or consequences.

The cost associated with adoption as a dominant reason for not adopting ABC is worth exploring further. Two themes are prevalent in this investigation: the belief that technology adoption costs are unaffordable or not justified. Delving further into this line of reasoning suggests that it may result from a misplaced perception by non-adopters. ABC technology as software indicates a wide price range for different variants of ABC technology adoption, and could not be termed excessively costly and thus used to justify non-adoption (Fichman and Kemerer, 2002). With multi-million dollar annual turnovers, the cost argument relative to benefits received from costing accuracies is a less convincing proposition. It is because ABC technology can help with identifying product mix changes, pricing changes, process changes, and organization restructuring (Umble and Umble, 2000).

The traditional cost system’s simplicity continues to appeal to many management, including in Australian universities. Traditional cost methods are sensible for gathering and assembling costs but do not convert the expenses into accurate managerial information (Gunasekaran et al., 2005; Ismail, 2010). The traditional cost system has become a legacy system for institutions that wish to maintain the status quo (Velmurugan, 2010, p. 7), and ABC technology is a viable alternative replacement.

## Data availability statement

The original contributions presented in the study are publicly available. This data can be found here: https://doi.org/10.6084/m9.figshare.21619578.

## Ethics statement

The studies involving human participants were reviewed and approved by Charles Darwin University Australia Human Research Ethics Committee approved this research (H16118). The ethics committee waived the requirement of written informed consent for participation.

## Author contributions

IA reconceptualized data, then revised, and rewrote the entire manuscript until journal publication. RS collected research data and wrote the first draft before data reconceptualization. All authors contributed to the article and approved the submitted version.

## Conflict of interest

The authors declare they conducted the research in the absence of any commercial relationships that could be construed as a potential conflict of interest.

## Publisher’s note

All claims expressed in this article are solely those of the authors and do not necessarily represent those of their affiliated organizations, or those of the publisher, the editors and the reviewers. Any product that may be evaluated in this article, or claim that may be made by its manufacturer, is not guaranteed or endorsed by the publisher.
